# Chemometric Studies on Natural Products as Potential Inhibitors of the NADH Oxidase from *Trypanosoma cruzi* Using the VolSurf Approach

**DOI:** 10.3390/molecules15107363

**Published:** 2010-10-21

**Authors:** Luciana Scotti, Elizabeth Igne Ferreira, Marcelo Sobral da Silva, Marcus Tullius Scotti

**Affiliations:** 1 LTF, University Federal of Paraíba, Campus I, João Pessoa-PB, Brazil; E-Mail: luciana.scotti@gmail.com (L.S.); 2 LAPEN, Department of Pharmacy, Faculty of Pharmaceutical Sciences, University of São Paulo - USP, São Paulo, SP, 05508-900, Brazil; E-Mail: elizabeth.igne@gmail.com (E.I.F.); 3 Centro de Ciências Aplicadas e Educação Campus IV - Litoral Norte, University Federal of Paraíba, Brazil

**Keywords:** natural products, VolSurf, antichagasic agents

## Abstract

Natural products have widespread biological activities, including inhibition of mitochondrial enzyme systems. Some of these activities, for example cytotoxicity, may be the result of alteration of cellular bioenergetics. Based on previous computer-aided drug design (CADD) studies and considering reported data on structure-activity relationships (SAR), an assumption regarding the mechanism of action of natural products against parasitic infections involves the NADH-oxidase inhibition. In this study, chemometric tools, such as: Principal Component Analysis (PCA), Consensus PCA (CPCA), and partial least squares regression (PLS), were applied to a set of forty natural compounds, acting as NADH-oxidase inhibitors. The calculations were performed using the VolSurf+ program. The formalisms employed generated good exploratory and predictive results. The independent variables or descriptors having a hydrophobic profile were strongly correlated to the biological data.

## 1. Introduction

The use of drug substances derived from plants, associated to their derivatives and synthetic compounds deduced from natural product precursors, represent a major part of today’s pharmaceutical market. Natural products provide opportunities in drug discovery, leading to a detailed understanding of biological pathways and revealing the functions of involved enzymes or receptors. The inhibition of NADH-oxidase and others mitochondrial enzyme systems may be an underlying mechanism for cytotoxicity and other biological effects of natural products [1,2,3,4,5].

Flavonoid compounds and analogues are naturally present in vegetables, fruits, and beverages and they are considered important components of the daily Western diet. They are also common constituents of medicinal plants, and the therapeutic effects of many traditional medicines have been attributed to these phytochemicals. These compounds exert distinct biological effects, particularly, acting as antioxidants and prophylactic agents against several diseases, including Chagas’ disease [1,2,3,4,5].

**Figure 1:**
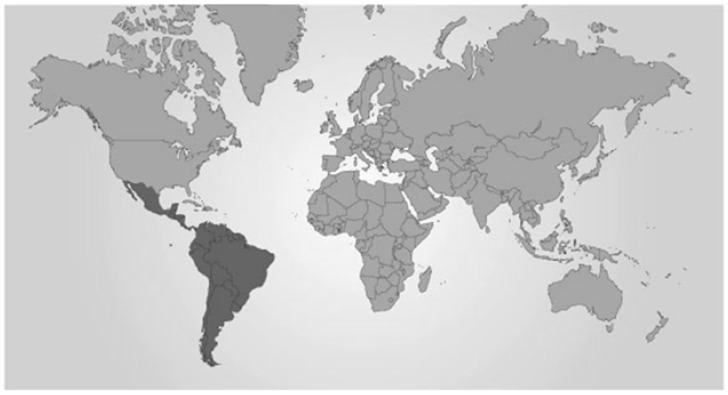
Geographic distribution of Chagas’ disease.

Chagas' disease (also called American trypanosomiasis) is a human tropical parasitic disease which occurs in the Americas, particularly in South America (Figure 1) [6]. The big social problem caused by this infection, in addition to the restricted number of drugs available and their serious side effects, as well as the emergence of new drug resistant forms, support the research for new antiprotozoal drugs.

**Figure 2:**
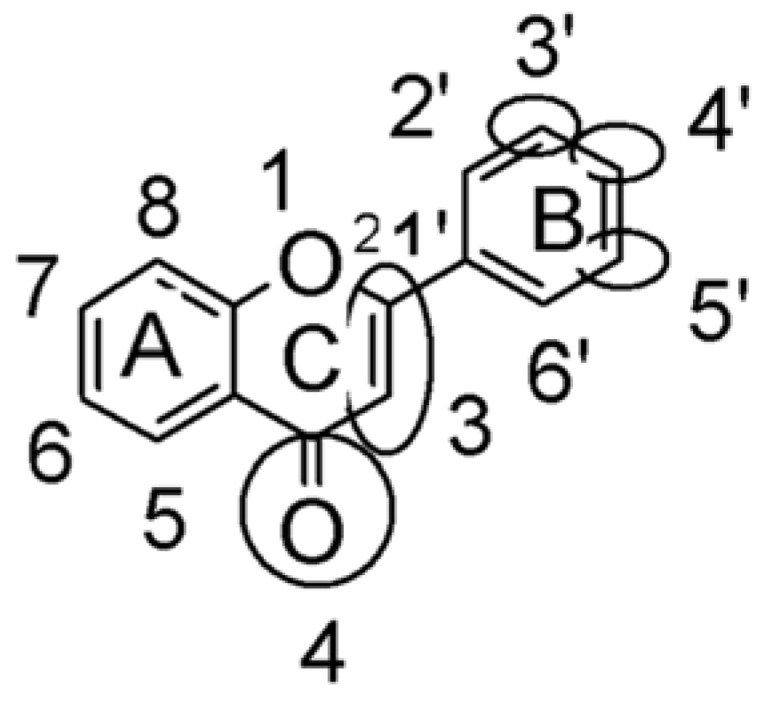
Chemical structure of flavones.

In several structure-activity studies, flavonoids have been tested considering their ability to inhibit key enzymes in *T. cruzi* mitochondrial respiratory pathway. The regions highlighted in the structure (see Figure 2) are: C2,3-double bond, C4-keto group and 3’,4’,5’-trihydroxy-B-ring, which are significant chemical features for those natural products are able to present a strong inhibition of NADH-oxidase [1,2,3,4], a potential key enzyme of mitochondrial respiratory pathway in *T*. *cruzi.*

Based on previous computer-aided drug design (CADD) studies [7,8] and regarding reported data on structure-activity relationships (SAR), an assumption concerning the action mechanism of natural products in parasitic infections was formulated, and it probably involves the T. cruzi NADH-oxidase inhibition. CADD methodologies associated to chemometric tools might be helpful to choose the most promising drug candidates.

Consensus PCA (CPCA) [9], Principal Component Analysis (PCA) and Partial Least Squares (PLS) regression are chemometric tools used for extracting and rationalizing the information from any multivariate description of a biological system. CPCA and PCA are part of an exploratory data analysis where graphical techniques provide a maximization of insights into a data set, pointing out important variables, detecting outliers and anomalies, and developing parsimonious models [10,11,12,13,14].

In this study, it was investigated a set of forty natural compounds, including flavonoids, flavonols, chalcones, diterpenes, isoflavones, and catechin, which are inhibitors of the *T. cruzi* NADH-oxidase. Molecular properties from 3D molecular fields of interaction energies (GRID approach) as well as the correlation of 3D molecular structures with physicochemical and pharmacokinetic properties were calculated. Chemometric tools as CPCA, PCA, and PLS regression were used to treat the resulting data, employing the program VolSurf+ [10,11,12,13,14].

## 2. Results and Discussion

### 2.1. CPCA

**Figure 3:**
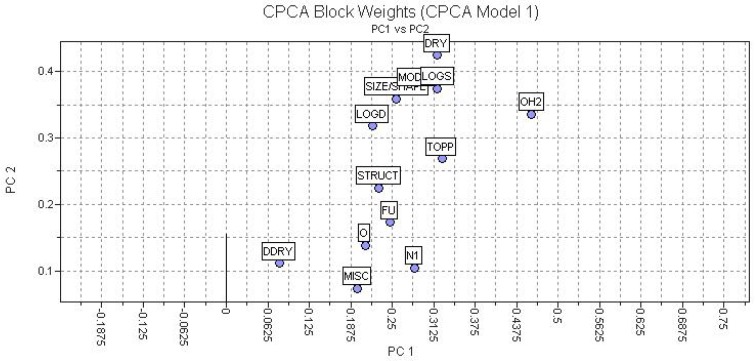
Plot of block weights considering PC or factor 1 and 2.

A preliminary exploratory analysis, CPCA, considering 128 independent variables or descriptors was developed. The preprocessing was performed (autoscaling), and 13 blocks of descriptors were calculated. Regarding Table 2, PC1 and PC2 explained a cumulative of 71.23% of total variance from the original data. The block formed by H2O (W1-W8, CW1-CW8, IW1-IW4) and DRY (D1-D8, CD1-CD8, ID1-ID4) descriptors had higher weights as presented in Figure 3.

### 2.2. PCA

**Figure 4:**
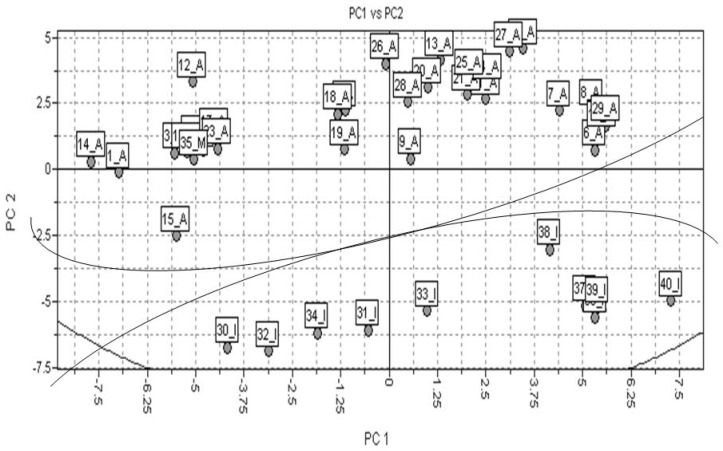
Scores plot from PCA, where active compounds, compounds having medium activity, and inactive compounds are represented as A, M, and I, respectively.

The PCA results were obtained regarding the interaction of 3D structures and a GRID force field, using the H2O and DRY probes. Forty molecular descriptors were calculated. The data were autoscaled (preprocess). Observing Table 3, PC1 and PC2 explained 76.55% of total variance from the original data. The scores plot showed a good discrimination between active (A), medium (M) and inactive (I) class of compounds, as presented in Figure 4.

### 2.3. PLS

**Figure 5:**
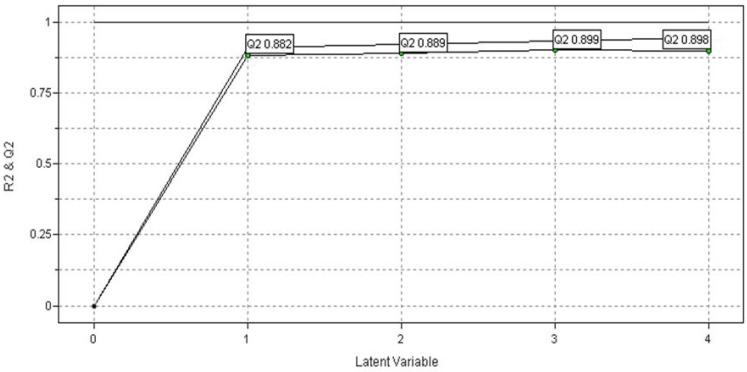
Plot of *r^2^* and *q^2^ versus* the number of latent variables (LV) considering the PLS models.

The training set is composed by thirty compounds and the test set is constituted of ten compounds (see Table 1), rationally selected as previously reported in Golbraikh *et al*. [28]. The autoscaling preprocess was also applied to the PLS discriminant analysis. The PLS analysis using the VolSurf descriptors as the X-block data and the NADH-oxidase inhibition values as dependent variables or Y-block generated significant statistical measures (leave-one-out cross-validation correlation coefficient, q_cv_^2^ = 0.899; and regression correlation coefficient, r^2^ = 0.931) when interactions fields were calculated using water and hydrophobic probes (see Figure 5). The maximum q^2^ value (0.899) was obtained using three latent variables (LV). Figure 5 shows that models containing four LVs presented an increment in r^2^ value, but the q^2^ value began to decrease. The model generated with three LVs explained 86.61% of total variance from the original data (see Table 4).

**Figure 6:**
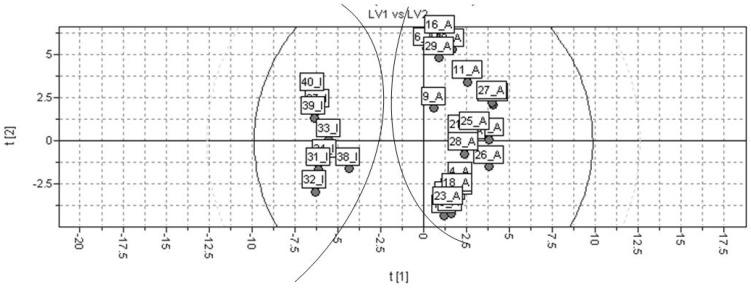
Discriminant PLS t1-t2 scores plot for the global model (A = active; I = inactive).

**Figure 7:**
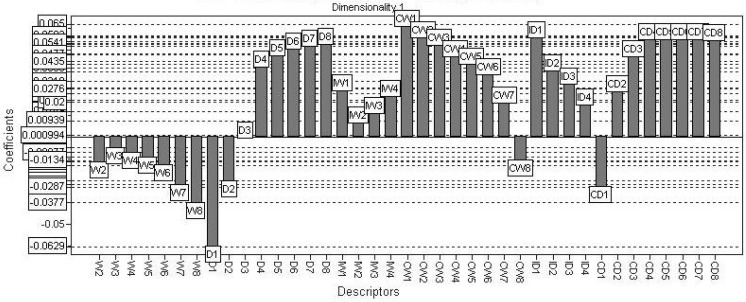
Coefficients plot generated from the selected PLS model.

The PLS t1-t2 scores plot of the resulting model is shown in Figure 6. Regarding the figure, the selected model provides a good discrimination between active and inactive class of compounds. The PLS coefficients found for the calculated VolSurf descriptors considering the global model (training and test sets combined) are presented in Figure 7. The coefficients plot indicates that variables presenting a hydrophobic profile, such as W1-8, CD1-8, CW1-8, D1-8, IW1-4, and ID1-4, have higher influence in the *T. cruzi* NADH-oxidase inhibition.

The external predictability (r^2^_ext_ = 0.703) was calculated using a test set containing ten compounds (7, 12, 14, 15, 19, 24, 30, 35, 36 – see Table 1). The active and inactive compounds were also perfectly distinguished.

### 2.4. Discussion

The claim used was an assumption regarding the mechanism of action of natural products against parasitic infections was formulated and involves the NADH-oxidase inhibition, a new hypothesis. The VolSurf descriptors were obtained from the interaction with water and hydrophobic probes calculated for all the molecules [10,12].

Regarding the CPCA formalism, a hundred and twenty-eight independent variables were taken into account and no biological data was given as input to the model. The orthogonal properties of CPCA algorithm were explored. The use of CPCA in decentralized process monitoring and diagnosis is derived in terms from the regular PCA scores and residuals. Two significant principal components (PCs) were found by a cross-validation technique, explaining about 75% of the total variance from original data (Table 2).

In CPCA we observed the super block-weights and, the importance of the each block has an influence in the calculations by comparing several blocks of descriptor variables measured on the same objects. Thirteen blocks of descriptors were calculated and their weights were plotted considering two factors: PC1 and PC2. Summarizing the observations in Figure 3, the DRY and H2O blocks presented significant weights in relation to PC2 and PC1. As already mentioned, the CPCA algorithm is basically equivalent to the regular PCA, but new definitions of block and variable of larger contributions were investigated in PCA and PLS.

The next step was the PCA method, where the 3D interaction energies calculated employing DRY and H2O probes in a GRID force field were considered, The PCA method was also applied to refine the data. The total number of descriptors calculated was forty. The findings generated by PCA were quite significant. PC1 and PC2 capture about 75% of the total variance from original data, using the leave-one-out (LOO) cross-validation technique (Table 3). There was a good classification between active and inactive compounds (see Figure 4). Defined clusters of active and inactive compounds were observed when the DRY end H2O VolSurf descriptors were used. This result indicates a strong predictability for the model.

Then, the PLS regression were applied to construct models considering a training set of thirty compounds. A test set of ten compounds was used for external validation procedure. The test set compounds were randomly selected, but rationality was used to be certain that the set was representative regarding global activity and structural diversity (Table 1). The best model provided by PLS regression presented three LVs, r^2^ = 0.931, and q^2^_LOO_ = 0.899, reinforcing the quality of the generated physicochemical VolSurf descriptors and biological data used in this study. It was observed an increment of statistical indices up to three LVs. After that, even though the r^2^ value was increased, the q^2^ value began to decrease (Figure 5). The model selected indicated a good discrimination between the active and inactive compounds (Figure 6).

The PLS scores plot demonstrates a quite good discrimination between highly and weakly active compounds in accordance to the significant statistical quality of the derived PLS model. In addition, that plot shows a very strong prediction power regarding the seven of the ten molecules from test set. The external predictability (r^2^_ext_ = 0.703) was calculated using a set of 10 compounds, which were not considered in the model construction.

The VolSurf descriptors having a relevant impact in inhibiting NADH-oxidase are highlighted in the PLS coefficients plot (Figure 7). According to the loadings plot, those descriptors are the following: W1-8, CD1-8, CW1-8, D1-8, IW1-4, and ID1-4.

The variable W1-8 describes the molecular envelope which is accessible to and attractively interacts with water molecules. The volume of this envelope varies with the level of interaction energies. Hydrophilic descriptors computed from molecular fields of -0.2 to -1.0 kcal/mol account for polarizability and dispersion forces; descriptors from molecular fields of -1.0 to -6.0 kcal/mol account for polar and H-bond donor-acceptor regions [10].

CD1-8 represents the ratio of the hydrophobic surface over the total molecular surface. It is the hydrophobic surface per surface unit [10]. CW1-8 represents the ratio of the hydrophilic surface over the total molecular surface. In other words, it is the hydrophilic surface per surface unit. D1-8 uses a probe called DRY to generate 3D lipophilic fields. In analogy to hydrophilic regions, hydrophobic regions may be defined as the molecular envelope generating attractive hydrophobic interactions [10].

IW1-4 and ID1-4 express the unbalance between the centre of mass of a molecule and the barycentre of its hydrophilic or hydrophobic regions [10]. When referring to hydrophilic regions, integy moments (IW1-IW4) are vectors pointing from the centre of mass to the centre of the hydrophilic regions: high integy moments indicate a clear concentration of hydrated regions in only one part of the molecular surface, small indicate that the polar moieties are either close to the centre of mass or they balance at opposite ends of the molecule, so that their resulting baricentre is close to the centre of the molecule. When referring to hydrophobic regions, integy moments measure the unbalance between the centre of mass of a molecule and the baricentre of the hydrophobic regions [10].

A hydrophobic tendency in the most active compounds was observed, mainly because the positive correlation coefficients of D3 and D8 descriptors, whereas the hydrophilic profile of W1-8 contributes negatively to the biological activity. However, hydrophilic surfaces seem to be also favorable.

Considering the positive coefficients found for the capacity factor variables (CW - H2O and CD – DRY), some areas strongly hydrophilic, as well as hydrophobic, are desirable to increase the inhibitory ability of the ligands.

It is important that the molecular surface should not be homogeneous. The ratio between the hydrophilic and hydrophobic surfaces, and the total molecular surface gives a positive and high value of the capacity factor variable (C). If the unbalance among the hydrophilic/hydrophobic areas in relation to the total surface increases, the contribution of the descriptor will be more positive to the activity. Observing the corresponding coefficients in the second PLS dimension plot, it was possible to deduce that ligands having a hydrophobic profile present a greater influence in inhibition of NADH-oxidase.

## 3. Experimental Section

In this study a set of forty natural compounds, including: flavonoids, flavonols, chalcones, diterpenes, isoflavones, and catechin, acting as NADH-oxidase inhibitors (Table 1), were selected from refs [1,2,3,4,5]. Biological activities were measured as the concentration required for 50% inhibition of NAOH-oxidase from beef heart [15]. The 50% inhibitory concentration of the investigated compounds were converted to molar units and then expressed in negative logarithmic units, pIC_50_ (-log IC_50_). The pIC_50_ values are given in Table 4 and comprise the set of dependent variables in this study. The range in activity for the analogues in Table 1 is about 6 (4.72–10.82) pIC_50_ units. In PLS, the models were constructed considering a training set of thirty compounds and a test set containing 10 compounds (see Table 1).

We studied the homology of NADH- oxidase between the beef heart protein and microorganisms. This mitochondrial enzyme is in cytochrome *bc1*. The cytochrome *bc1* complex is an oligomeric membrane protein complex which transfers electrons from a relatively low-potential quinol to a c-type cytochrome with a high degree of homology (≈ 85%) of the *bc1* subunits [16].

Compound **35** is rotenone, considered a potent inhibitor. Rotenone is used in solution as a pesticide and insecticide, or in emulsified liquid form as a pesticide. It works by interfering with the electron transport chain in mitochondria, inhibits the transfer of electrons from iron-sulfur centers in complex I to ubiquinone. It inhibits the NADH-oxidase interfering in the electron transportation throughout the respiratory path at mitochondria. However, in this work, it was classified as having a medium activity when compared to all flavonoids and other analogues, which are more potent as NADH-oxidase inhibitors.

The three-dimensional structures of each forty analogues in their neutral forms were constructed using the HyperChem 6.0 software [17]. The following crystallized structures were retrieved from Protein Data Bank (PDB) and their ligands were used as starting geometries for building up the 3D models of the investigated set: 1fm8 (2.30 Å resolution) [18], 2brt (2.20 Å resolution) [19], 1gp5 (2.20 Å resolution) [20], 1eyq (1.85 Å resolution) [21], 1fm7 (2.30 Å resolution) [18], and 1jep (2.10 Å resolution) [20]. Each ligand model was energy-minimized employing the HyperChem 6.0 MM+ force field without any restriction [22,23], and partial atomic charges were assigned using the AM1 [24] semiempirical method, also implemented in the HyperChem program.

The structures modeled as described above were used as the initial structures to calculate the molecular descriptors employing the VolSurf+ program [25]. PCA, CPCA and PLS methodologies were applied to the investigated set using the VolSurf+ software [26,27].

**Table 1:** Structures and biological activities of the forty investigated compounds. ^a^

Name [*Id*]	Biological activity pIC_50_	Chemical structure
5 – Hydroxyflavone [*1*]	9.37^3^	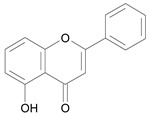
7,8 – Dihydroxyflavone [*2*]	9.56^2^	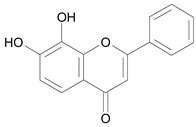
7 – Hydroxyflavone [*3*]	8.85^3^	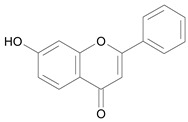
Apigenin [*4*]	9.04^3^	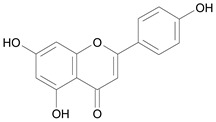
Baicalein [*5*]	10.11^3^	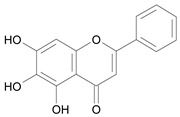
Butein [*6*]	10.74^1^	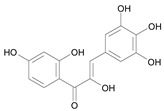
(2*R*,3*S*) – Catechin [*7*]	8.74^1^	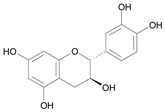
(2*S*,3*R*) – Catechin [*8*]	8.74^1^	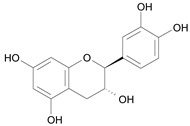
Cyanidin [*9*]	9.30^1^	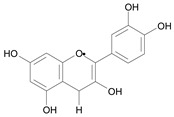
Crysin [*10*]	9.60^3^	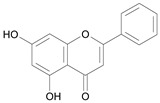
Delphidin [*11*]	9.00^1^	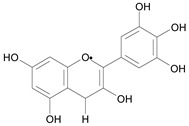
Eupatorin [*12*]	10.37^2^	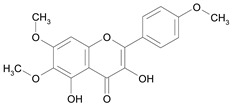
Fisetin [*13*]	10.82^1^	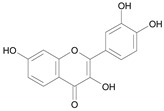
Flavone [*14*]	9.62^3^	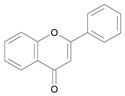
Flavanone [*15*]	9.51^3^	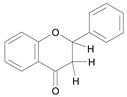
Fustin [*16*]	9.90^1^	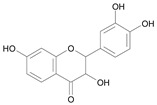
Galangin [*17*]	8.72^1^	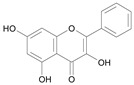
Genistein [*18*]	9.44^3^	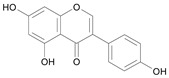
Kaempferol [*19*]	8.72^1^	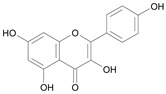
Luteolin [*20*]	10.32^1^	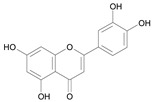
Morin [*21*]	9.37^1^	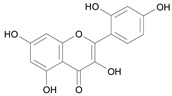
Myrecitin [*22*]	10.46^1^	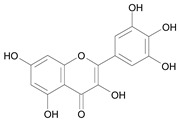
Norwogonin [*23*]	9.47^2^	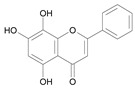
Quercetagin [*24*]	9.75^1^	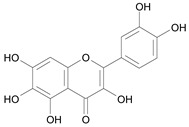
Quercetin [*25*]	9.84^1^	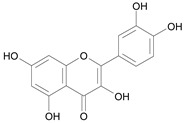
Rhamnetin [*26*]	7.38^2^	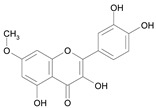
Robinetin [*27*]	7.72^2^	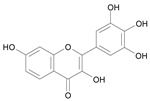
Rutin [*28*]	8.72^3^	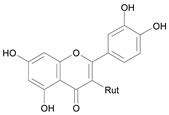
Taxifolin [*29*]	9.76^1^	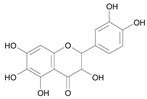
Tigliane 1 [*30*]	5.60^4^	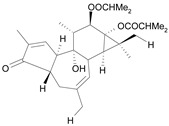
Tigliane 2 [*31*]	4.72^4^	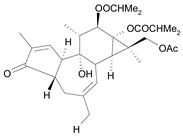
Tigliane 3 [*32*]	4.72^4^	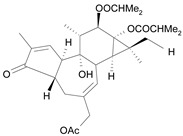
Tigliane 4 [*33*]	4.88^4^	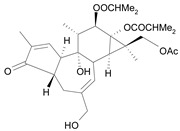
Tigliane 5 [*34*]	5.11^4^	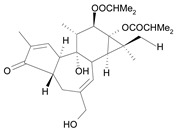
Rotenone [*35*]	8.29^4^	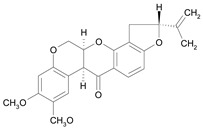
Jatrophane 1^b^ [*36*]	5.20^5^	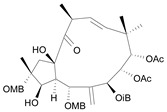
Jatrophane 2 ^b^ [*37*]	5.29^5^	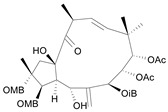
Jatrophane 3 ^b^ [*38*]	5.15^5^	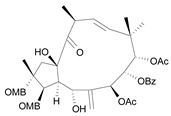
Jatrophane 4 ^b^ [*39*]	4.86^5^	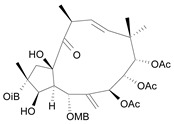
Jatrophane 5 ^b^ [*40*]	4.96^5^	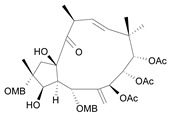

^a^ The NADH oxidase assay were monitored by a modified manometric procedure at 30 ºC for flavonoids and analogues and 22 ºC for diterpenes for their ability to inhibit beef heart mitochondrial NADH-oxidase activity. The test set comprises the compounds **7**, **12**, **14**, **15**, **19**, **24**, **30**, **35**, **36**. The other 30 compounds constitute the training set; ^b^ Ac = acetate, MB = 2-methylbutyrate, iB = isobutyrate , Bz = benzoate.

## 4. Conclusions

The chemometric tools applied in this study generated good exploratory and predictive results. The significant results from CPCA, PCA prediction and PLS discriminant models can be helpful for designing new antichagasic agents acting as NADH-oxidase inhibitors. The VolSurf descriptors showed that the presence and the unbalance of the hydrophilic profile in relation to the total molecular surface, and also a hydrophobic profile, are strongly correlated to the biological data.

## Figures and Tables

**Table 2:** Variance explained by CPCA.

PC	% explained variance from original data
1	38.99
2	32.24
3	7.53
4	3.87
5	2.52

**Table 3:** Variance explained by PCA.

PC	% explained variance from original data
1	43.72
2	32.83
3	10.02
4	5.95
5	2.07

**Table 4:** Variance explained PLS models.

LV	% explained variance from original data
1	32.08
2	36.99
3	17.54
4	3.34
